# Lithium intoxication related multiple temporary ecg changes: A case report

**DOI:** 10.1186/1757-1626-1-156

**Published:** 2008-09-17

**Authors:** Fatih Canan, Ahmet Kaya, Serkan Bulur, Enver Sinan Albayrak, Serkan Ordu, Ahmet Ataoglu

**Affiliations:** 1 Duzce University, Faculty of Medicine, Department of Psychiatry, Duzce, Turkey; 2 Duzce University, Faculty of Medicine, Department of Cardiology, Duzce, Turkey

## Abstract

Lithium is a widely used mood stabilizer, which may cause cardiac side effects. In this article, we present the case of a 39-year-old woman who had presented with pre-syncope and developed multiple ECG abnormalities that are caused by lithium intoxication and are disappeared after hemodialysis.

## Introduction

Lithium salts are widely used in psychiatric therapy and prophylaxis as mood stabilizers. The mechanism as a mood-stabilizing agent remains unknown, although effects on biologic membranes are speculated. A serum level between 0.8 mEq/l and 1.2 mEq/l is considered therapeutic, usually obtained with a dose of approximately 900 to 1200 mg/day [1].

The cardiac side effects of lithium have been well described at a wide range of plasma concentrations [2,3]. Lithium salts may induce various electrocardiographic (ECG) changes including nonspecific T-wave flattening [2], dysfunction of sinus node [4-6], and prolonged QT interval [7,8]. In rare cases, ventricular tachycardia and ventricular fibrillation resulting in death have been reported [2].

Herein, we describe the case of a 39-year-old woman who had presented with pre-syncope and developed multiple ECG abnormalities that are caused by lithium intoxication and are disappeared after hemodialysis.

## Case report

A 39-year-old female patient with a history of bipolar disorder and mental retardation was admitted to emergency service with agitation and elevated mood. She had been using various mood stabilizers irregularly until she had discontinued her treatment (Valproic acid 1 gr/day) 3 months ago. The condition was diagnosed as "manic episode" according to DSM-IV-TR criteria and she was administered intramuscular ziprasidone 20 mg and started on lithium (1200 mg/day, 3 times per day).

After 5 days, she was admitted to emergency service again with the complaints of fainting, altered mental status, and motor weakness particularly of lower extremities. She was found to be unable to walk and follow simple commands. Her initial vital signs were normal (BP: 130/90 mmHg, HR: 52 beats/minute). Her laboratory findings were notable for hypokalemia with a potassium level of 2.72 mEq/l (normal range: 3.2–5.1), and hypophosphatemia (1.26 mEq/l) (normal range: 2.7–4.5). Her serum lithium level was 2.96 mEq/l (therapeutic level: 0.8–1.2), indicating lithium intoxication. She was hospitalized for hemodynamic support and urgent hemodialysis.

**Figure 1:**
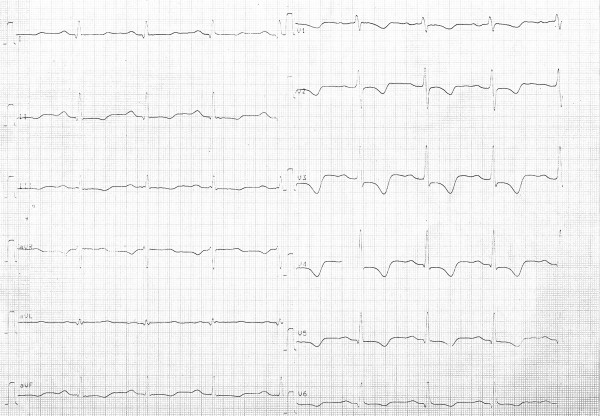
Twelve-Lead ECG at admission (PR: 320 msec, QRS: 120 msec, QT: 640 msec).

The patient's ECG (Figure 1) was remarkable for expanded p-wave (180 msec), expanded QRS (120 msec), prolonged QT (640 msec) and PR (320 msec) interval, ST wave depression in leads V2 and V3, and common T wave inversion (Figure 1).

**Figure 2:**
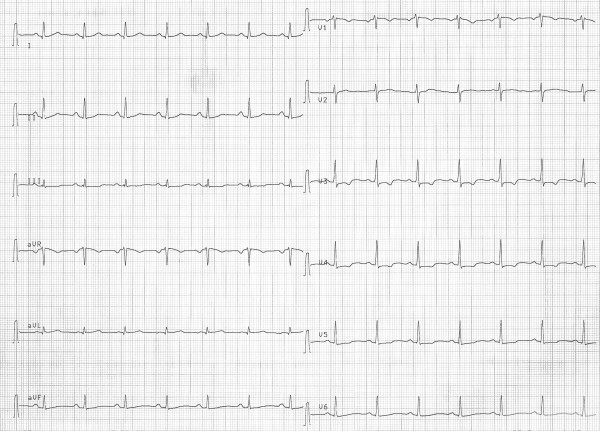
Twelve-Lead ECG after hemodialysis (PR: 150 msec, QRS: 60 msec, QT: 360 msec).

After hemodialysis, the patient's lithium level decreased to 0.57 mEq/l and her general condition improved rapidly. Her control ECG (Figure 2) revealed no abnormalities except T wave inversions and slight ST depression in leads V2 and V4. She was discharged from the hospital with risperidone 4 mg/day and recommended psychiatric follow-up.

## Discussion

Lithium salts are highly water-soluble and are well absorbed by the gastrointestinal tract. Complete absorption occurs in about 8 hours, with peak concentrations occurring 2 to 4 hours after an oral dose. Lithium is initially distributed in the extra cellular fluid and then accumulates gradually in various tissues. Approximately 95% of a single dose of lithium is eliminated in the urine; with repeated administration, the excretion increases until a steady state is reached after 5 to 7 days [9].

Animal experiments indicate that lithium depresses the intracellular potassium concentration. In addition lithium replaces intracellular calcium [10]. These disturbances seem to induce various electrophysiological changes including a decrease of the depolarization rate and reduced electrical impulse propagation [11]. It has also been suggested that lithium might decrease the sensitivity of the sinus node to sympathetic stimulation [12]. Interaction between lithium and several currents such as IK, ICa, the Na/Ca exchange current, and a Na/K pump current might lead to the observed results [13]. It is indefinite whether cardiac effects in patients receiving lithium are caused by lithium toxicity, or are a co-incidental finding. The two appear to be causally linked, as indicated by our case report.

The clinical features and optimal management of lithium intoxication have recently been reviewed [14]. This indicates that haemodialysis is generally more effective in lithium clearance than other modalities, for example, haemofiltration. However, it appears that prolonged haemodialysis (> 16 hours) is required in order to prevent 'rebound' of lithium concentrations. Prolonged hemodialysis appears necessary to allow adequate tissue removal of lithium. Although we did not perform single-day prolonged hemodialysis, we accept it as a logical way of treatment since we had to perform four days of serial hemodialysis consecutively in our patient in order to decrease lithium level to therapeutic range.

A key area of importance of our case report is that our patient developed severe clinical features, whereas lithium concentrations were only modestly elevated. This is the main characteristic of chronic lithium poisoning, as described by Waring et al. [15]. Lithium concentrations > 5 mmol/L might be used to indicate a need for haemodialysis after acute lithium overdose, whereas, a much lower treatment threshold is recommended for chronic toxicity, or acute-on-therapeutic toxicity (and the clinical features are much more important guide than blood levels for chronic poisoning).

The uses of lithium continue to expand with an increasingly larger population. This case report shows that lithium toxicity can occur even in therapeutic dosages particularly when combined with other medications such as ziprasidone.

Clinicians should be vigilant to cardiac risks and ECG changes associated with lithium toxicity, including all kinds of arrhythmias that can occur abruptly in the first days of the treatment.

## Competing interests

The authors declare that they have no competing interests.

## Authors' contributions

FC, AK, SB conceived the case report. FC, SO, ESA prepared the manuscript. FC, ESA, AA finalised the manuscript. All authors read and approved the final manuscript.

## Consent

Written informed consent was obtained from the patient for publication of this case report and any accompanying images. A copy of the written consent is available for review by the Editor-in-Chief of this journal.

## References

[B1] Griswold KS, Pessar LF (2000). Management of bipolar disorder. Am Fam Physician.

[B2] Mitchell JE, Mackenzie TB (1982). Cardiac effects of lithium therapy in man: a review. J Clin Psychiatry.

[B3] Waring WS (2007). Delayed cardiotoxicity in chronic lithium poisoning: discrepancy between serum lithium concentrations and clinical status. Basic Clin Pharmacol Toxicol.

[B4] Goldberger ZD (2007). Sinoatrial block in lithium toxicity. Am J Psychiatry.

[B5] Riccioni N, Roni P, Bartolomei C (1983). Lithium-induced sinus node dysfunction. Acta Cardiol.

[B6] Rosenqvist M, Bergfeldt L, Aili H, Mathé AA (1993). Sinus node dysfunction during long-term lithium treatment. Br Heart J.

[B7] Mamiya K, Sadanaga T, Sekita A, Nabeyama Y, Yao H, Yukawa E (2005). Lithium concentration correlates with QTc in patients with psychosis. J Electrocardiol.

[B8] Mateer JR, Clark MR (1982). Lithium toxicity with rarely reported ECG manifestations. Ann Emerg Med.

[B9] Baldesarini RJ, Tarazi FI, Hardman JG, Limbird LE (2001). Drugs and the treatment of psychiatric disorders. Psychosis and mania. Goodman & Gilman's The pharmacological basis of therapeutics.

[B10] El-Mallakh RS (1990). The ionic mechanism of lithium action. Lithium.

[B11] Singer I, Rotenberg D (1973). Mechanism of lithium action. N Eng J Med.

[B12] Fann WE, Davis JM, Janowsky DS, Cavanaugh JH, Kaufmann JS, Griffith JD, Oates JA (1972). Effects of Lithium on adrenergic function in man. Clin Pharmacol Ther.

[B13] Irisawa H, Gilles WR, Zipes DP, Jalife J (1990). Sinus and atrioventricular node cells: cellular electrophysiology. Cardiac electrophysiology From cell to bedside.

[B14] Waring WS (2006). Management of lithium toxicity. Toxicol Rev.

[B15] Waring WS, Laing WJ, Good AM, Bateman DN (2007). Pattern of lithium exposure predicts poisoning severity: evaluation of referrals to a regional poisons unit. QJM.

